# Antilisterial activity of *Cymbopogon citratus* on crabsticks

**DOI:** 10.3934/microbiol.2018.1.67

**Published:** 2018-02-02

**Authors:** Prateebha Ramroop, Hudaa Neetoo

**Affiliations:** Department of Agricultural and Food Sciences, Faculty of Agriculture, University of Mauritius, Mauritius

**Keywords:** lemon grass, *Listeria monocytogenes*, *Listeria innocua*, surimi, sensory, pH

## Abstract

*Listeria monocytogenes* is a gram positive, psychrotrophic, facultative anaerobic bacterium and it is the etiological agent of listeriosis, a severe foodborne disease of major public health concern. There is a rising concern about the cross-contamination of surimi-based products with *L. monocytogenes* during handling and storage. Lemon grass (*Cymbopogon citratus*) is known to exhibit strong antimicrobial activity against bacteria due to the presence of citral. The objectives of this research were: (i) to develop a water-based extraction procedure for the antimicrobial component(s) in lemon grass and (ii) to evaluate the antimicrobial effect of a concentrated water-based extract and commercial essential oil (EO) of lemon grass against *L. innocua* (ATCC 33090), a surrogate strain of *L. monocytogenes*, *in vitro* and on crabsticks. Briefly, antilisterial activity of concentrated extract and commercial EO of lemon grass was tested using the agar well diffusion technique. Crabsticks were subsequently inoculated with *L. innocua* to a final density of ca. 4 log cfu/g and then coated with 500 μl of either concentrated extract or 0.5% commercial EO and stored at 4 °C for up to 15 days. Samples were then subjected to microbiological analysis every 5 days to enumerate counts of *Listeria*. Following the agar well diffusion assay, inhibition zones with mean diameters of 18.3 and 21.0 mm were obtained with the concentrated extract and commercial EO respectively. The population of *L. innocua* in WBE-coated (4.2 log cfu/g) and 0.5% EO-coated (2.7 log cfu/g) samples were significantly lower (*P* < 0.05) after 15 days than their untreated control counterpart (5.2 log cfu/g). Lemon grass extract and essential oil have the potential to control growth of *L. monocytogenes* in seafood surimi products with minimal adverse effect on the organoleptic characteristics of the product and thus can possibly be used as a natural food preservative.

## 1. Introduction

Ready-to-eat (RTE) seafood products are becoming more popular and have a high demand mainly due to their convenience. They are low in calories and have a low fat and cholesterol content, thereby contributing negligibly to weight gain. They also have a high nutritional value since they are rich in protein and Omega-3 fatty acids. Imitation crabstick is a ready-to-eat seafood made with fish surimi. Unfortunately, contamination of ready-to-eat foods by *Listeria monocytogenes* poses a considerable safety concern. This ubiquitous bacterium can grow over a wide pH range of 4.1 to around 9.6, at a temperature range of 1 to 45 °C and at high salt concentrations of up to 10% [1]. RTE seafood products are frequently contaminated with *L. monocytogenes* due to post-process recontamination in the processing plant. Since they are often eaten without any final heat-killing step, they can constitute a food hazard. To date, the minimum infective dose of *L. monocytogenes* is still unknown and therefore a “zero tolerance” policy for presence of *L. monocytogenes* in RTE foods is enforced in the United States by both the Center for Food Safety and Applied Nutrition (CFSAN) and USDA Food Safety and Inspection Service (FSIS). In Mauritius, there are currently no regulations governing the presence of *L. monocytogenes* in RTE seafood products. However, application of additional post-processing hurdles such as treatment with natural antimicrobials is still highly recommended to inhibit the growth of *L. monocytogenes*.

Developing more environment-friendly and harmless products of natural origin as an alternative to synthetic food preservatives such as sodium nitrite and potassium sorbate has been the focus of research lately due to the fact that consumers are becoming more aware of the side effects of the latter. Furthermore, the World Health Organization has called for a worldwide decrease in salt consumption so as to reduce the probability of cardiovascular diseases to occur thus other additives are needed to ensure food safety. Plant essential oils are effective and promising natural antimicrobials and can be used as alternatives to synthetic preservatives [2]. Between 400 and 500 essential oils (EO) are being commercially produced [3] and lemongrass (LG) essential oils of the *Cymbopogon* species have gathered significant scientific interest. Previous studies have shown that whole lemon grass EO has an antimicrobial effect on a range of microorganisms including pathogens [4]–[6]. Citral is the major component of LG essential oil (65–85%) [7]. *C. citratus* has a strong antimicrobial activity due to citral and its effect on gram-positive bacteria is greater than gram-negative bacteria [8]. Water-based extracts (WBE) of lemon grass can also be obtained through a maceration method. Commercially available lemon grass EO and WBE can be subsequently added to food products to enhance their microbiological safety and quality. The aim of this study was to assess the antimicrobial effect of *Cymbopogon citratus* (lemon grass) against *Listeria innocua*, a surrogate organism for *L. monocytogenes*, on a model food system. Specifically, the objectives were to: (i) Extract the bioactive component of *Cymbopogon citratus* using water as solvent. (ii) Assess the antimicrobial property of lemon grass WBE and LG commercial essential oil at different concentrations against *Listeria innocua* *in vitro* and on crabsticks and finally. (iii) Assess the effect of the aforementioned treatments on the physico-chemical characteristics such as color measurement and pH.

## 2. Materials and methods

Lemon grass plants (*Cymbopogon citratus*) were collected during the months of October to January from the North Eastern region of Mauritius. The accession number of the plant samples was authenticated at the herbarium of the Mauritius Cane Industry Authority and identified as MAU0022470. Lemon grass essential oil was bought from a local pharmacy found in the North East region of Mauritius. *Listeria innocua* (ATCC 33090), a surrogate strain of *Listeria monocytogenes*, was obtained from microbiology laboratory of the Faculty of Agriculture, University of Mauritius. Surrogate microorganisms are harmless microorganisms, which have similar resistance properties to pathogenic organisms and can be used as substitutes for testing. Since *Listeria monocytogenes* is pathogenic and there are certain biosafety restrictions in its handling, surrogate strain *Listeria innocua* ATCC 33090 [9] has been selected for this study. In using this surrogate, the assumption has been made that it would respond similarly as the pathogenic strain *Listeria monocytogenes*. Gram staining was done to confirm the identity of the strain. Crabsticks (Golden Champ) were bought at a supermarket located in North East of Mauritius.

### 2.1. Solvent extraction

Water-based extraction of lemon grass was done using maceration method [10]. It involves cutting lemon grass stems and leaves into pieces; and seeping 100 g in 250 mL of water in a conical flask. Cotton wool was used to plug the mouth of the conical flask, which was then wrapped with newspaper to minimize water loss due to evaporation. The conical flask was then placed on a mechanical shaker for 3 days. The composition was filtered using a pressure pump after 3 days, which was followed by a 0.2 µm membrane filtration to remove bacteria and impurities.

### 2.2. In-vitro antimicrobial susceptibility testing

Agar well diffusion technique was used [11]. About 100 mL of PALCAM agar supplemented with *Listeria* supplement was poured in sterile petri dishes and allowed to solidify. The agar was made thick enough so that wells could be punched easily. They were then inoculated with 100 µl of a late-log phase culture of *Listeria innocua* by spread plate technique. Six holes were punched aseptically using a 6-mm diameter sterile cork borer. Hundred microliters of 0.5%, 0.2%, 0.1%, 0.05% and 0.01% of the LG essential oil was added in each respective labeled well and 100 µl of 30% ethanol was added into one well representing a negative control. A total of three wells were also punched for testing the water-based concentrate and a two-fold dilution as well as a negative control (sterile water) by aliquoting 100 µl in each of these wells. The petri dishes were then incubated at 35 °C for 48 hours. Triplicates were done for each treatment. Zones of inhibitions were then measured using a ruler and compared.

### 2.3. Antimicrobial testing in a food system

The antilisterial activity of the lemongrass EO (0.5%) as well as the pure water-based extract (WBE) was compared. Briefly, a loopful of *Listeria innocua* growth on PALCAM agar was inoculated in a test tube containing 10 ml of peptone water, which was then incubated at 35 °C for 48 hours to reach a late-log phase. The crabsticks were inoculated with a 500-µl aliquot of dilution of the culture (7 log cfu/ml) on each side to a final population density of approximately 4 log cfu/g. These sticks were then allowed to dry for about 5 min before subsequent treatments. 500 µl of either pure WBE, 0.5% essential oil of lemon grass, sterile water (solvent control for WBE) or 30% ethanol (solvent control for EO) were then applied onto each side of the surface. This was carried out in duplicates. Two crabsticks were also inoculated with *L. innocua* and left untreated (negative control). The crabsticks containing the several treatments were then vacuum-packaged (MULTIVAC) and kept at 4 °C for 15 days of storage. Microbial analysis of the crabsticks was done at an interval of 5 days. Crabstick weighing ca. 16 g was placed in a stomacher bag containing 64 ml of peptone water i.e., diluted 5-fold. The mixture was homogenized using a stomacher. The mother sample and its dilutions were plated on PALCAM agar and incubated at 35 °C for 48 hours for enumeration of listerial count. All the colonies were then enumerated with a colony counter. Putative colonies of *L. innocua* from inoculated crabsticks were compared with colonies from an overnight pure culture of the same strain on PALCAM agar. To ensure that *Listeria* recovered from inoculated crabsticks were not from the microbiota of the product itself, uninoculated crabsticks were microbiologically analysed on PALCAM agar to recover any endogenous listeriae.

### 2.4. Physicochemical analysis

Uninoculated crabsticks were similarly treated as described above i.e., they were coated with 500 µl of either pure water-based extract, 0.5% essential oil of lemon grass, sterile water (solvent control for WBE), or 30% ethanol (solvent control for EO) onto each side of the surface. Two crabsticks were also left untreated (negative control). The experiment was carried out in duplicates. The samples were analyzed at the beginning and end of the refrigerated storage period (Day 0 and Day 15) at 4 °C. For determination of pH, minced and crushed crabstick (16 g) was added in 64 mL of distilled water and a magnetic stirrer was used to stir it for 30 minutes. The pH of the crabsticks was then measured using a pH meter (Mettler Toledo). For determination of % drip water loss, crabsticks were weighed at the beginning and end of the storage period (Day 15). Instrumental surface colour (CIE L* a* b*) of both sides of the crabsticks was also determined by using a chromameter (Minolta CR-410, Konica Minolta, Japan). All the physicochemical analyses were carried out in two independent replicates and their measurements were taken in triplicates.

### 2.5. Sensory evaluation

Uninoculated crabsticks were similarly treated as described above i.e., they were coated with 500 µl of either pure water-based extract, 0.5% essential oil of lemon grass, sterile water (solvent control for WBE), or 30% ethanol (solvent control for EO) onto each side of the surface. A sensory evaluation questionnaire was designed and sensory analysis was conducted with 15 untrained panelists at the beginning (Day 0) and end (Day 15) of storage. On each day of the analysis, samples were taken out and rated for different sensory parameters such as colour, aroma, texture, general appearance or appeal and overall acceptability ranked on a scale of 1 to 5 where 5 represented the highest score and 1 the lowest.

### 2.6. Statistical design and analysis

All experiments were conducted in two independent trials. Where appropriate, statistical analyses were conducted using Minitab^®^ Release 18. *In vitro* test results were analysed using a single factor analysis of variance (ANOVA), and Tukey pairwise comparisons at 95% confidence interval were conducted to determine significant differences in inhibition zone diameters from the various treatments. Results for physico-chemical, sensorial and microbiological investigations of crabsticks were analysed using a generalized linear model (GLM) based on ANOVA model that included the effects of treatment and days of storage, as well as the interaction between treatment and days of storage. The post-hoc Tukey's method was applied for pairwise comparison. Statistical significance was attributed to *P* < 0.05.

## 3. Results and discussion

### 3.1. In vitro antilisterial activity

The average results obtained are shown in the Table 1 below. It can be observed that 0.5% commercial oil had a significantly (*P* < 0.05) greater zone of inhibition (ZOI) (21.0 mm) compared to either the pure WBE (18.3 mm) or the solvent controls.

**Table 1.** Mean and standard deviation of ZOI of the antimicrobials.

Treatments	Inhibition zone/mm
Water	0.0 ± 0.00^d^
Ethanol	6.3 ± 0.58^c^
Water-based extract	18.3 ± 1.52^b^
0.5% Commercial oil	21.0 ± 1.00^a^

Data represent mean values ± sd; Means with different superscripts differ significantly.

*In vitro* antilisterial activity of EO as determined by the well diffusion assay revealed inhibition zones of 21.0 mm which corroborates findings of Nguefack et al. [12] who also obtained comparable inhibition zone sizes (20.8 mm). Sharoba et al. [13] tested the antilisterial activity of lemongrass oil using the disk diffusion method and obtained inhibition zones of 14 mm against *Listeria monocytogenes* thereby showing a moderate activity. To the best of our knowledge, no previous studies have been done on the antilisterial activity of water-based extracts of lemongrass. In our study, we demonstrated that lemongrass WBE also exhibits appreciable antilisterial activity with zones of inhibition of 18 mm. WBE in this study was obtained using the maceration method. Unlike this study, other authors such as Balakrishnan et al. [14] have carried out water-based extraction of lemon grass using the soxhlet apparatus and reported a ZOI of 15 and 20 mm against gram-positive bacteria *Staphylococcus aureus* and *Bacillus subtilis* respectively when testing the extract (500 mg/ml).

However the results of the current study cannot really be compared with previous studies since there are many factors that differ such as the bacterial strain, extraction, assay method and chemical composition of lemon grass. We can infer from the study that lemon grass essential oil has slightly higher activity compared to the water-based extract possibly due to the differences in their composition. Indeed, non-polar or volatile compounds having a low polarity (neral and geranial) are usually present in essential oils whereas polar compounds are typically present in WBE [15]. EO is thought to have lightly larger inhibition zones than the WBE due to diffusion of its active components through the agar as well as volatility of the active constituents whereas the WBE can only diffuse through the agar [15]. Also the method used to extract essential oil by either hydrodistillation or soxhlet apparatus, and use of solvent such as ethanol or methanol could cause release of secondary metabolites from the plant sample compared to the mild water-based extraction. Using water, only anthocyanins, starches, tannins, saponins, terpenoids, polypeptides and lectins are extracted [16].

Citral, which is responsible for the antimicrobial action in lemongrass is a monoterpenoid aldehyde, often presented as neral and geranial which are stereoisomers. The strong activity of EO has a correlation with the high content of citral [17], which is itself bacteriostatic. Upon treatment with citral, the bacterial cell membranes become more permeable, with ensuing cell morphology changes and decreases in ATP synthesis leading to cl damage [18]. Cui et al. [19] demonstrated that listerial cells treated with lemongrass essential oil became rough and distorted compared to untreated cells, which remained smooth. In addition to citral, other oxygenated terpenes are also more present in *Cymbopogon citratus* than terpenes hydrocarbon. It is the oxygenated terpenes that mainly show the antimicrobial activity than terpenes hydrocarbon since the latter has a low hydrogen-bonding capability and is poorly soluble in water limiting its diffusion in the agar [20]. Myrcene also is a main component of LG oil (99%), but it does not show any observable antimicrobial effect though it appears to enhance the activity of citral [21]. The antimicrobial effect of lemongrass was also previously investigated by Sikkema et al. [22]. The author demonstrated that the antimicrobial effect is due to monoterpenes, which exerts toxic effects on the structure and function of the cell plasma membrane.

### 3.2. Anti-listerial effectiveness of treatments on a food system

**Figure 1.**
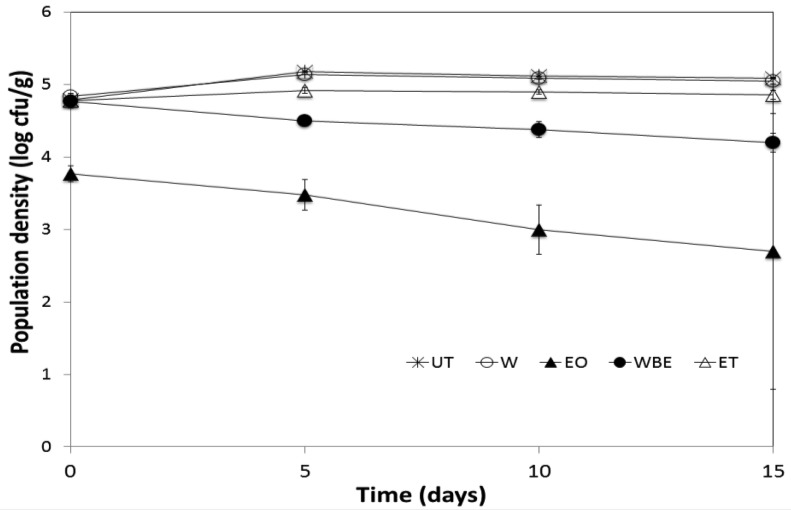
Mean population density of *Listeria* on crabsticks subjected to different treatments over a 15-day storage period.

The five treatments tested on crabsticks inoculated with *L. innocua* were UT (Untreated), W (Deionized water), EO (0.5% Lemon grass commercial essential oil), WBE (Water-based LG extraction) and ET (30% Ethanol). Figure 1 show that the counts of *L. innocua* increased in untreated crabsticks from 4.79 log cfu/g (Day 0) to 5.18 log cfu/g (Day 5) then decreased slightly to a final population of 5.09 log cfu/g. For W and ET-treated crabsticks, the counts also increased from 4.84 log cfu/g (Day 0) and 4.78 log cfu/g (Day 0) to 5.14 log cfu/g (Day 5) and 4.92 log cfu/g (Day 5) respectively then decreased at a small rate up to Day 15 although the final population was not less than the count on Day 0. For the EO- and WBE-treated crabsticks, the listerial count decreased continuously from 3.77 log cfu/g to 2.7 log cfu/g and from 4.77 log cfu/g to 4.2 log cfu/g, respectively. In fact, the treatment in question, the duration of storage as well as the interaction between the treatment and duration of storage was found to significantly (*P* < 0.05) affect the population density of *Listeria* on the product. In addition, a significant decrease (*P* < 0.05) of 1.02 log cfu/g was obtained between the untreated and EO-treated samples at Day 0, and a significant difference (*P* < 0.05) of 2.39 log cfu/g at Day 15. For WBE, the population of *Listeria* was only lower than their untreated counterparts by 0.02 log cfu/g (Day 0) and 0.89 log cfu/g (Day 15), respectively. The negative controls W and ET had counts comparable to the untreated sample. However a difference of 0.23 log cfu/g was obtained between ET and UT at Day 15 although the difference was not statistically significant (*P* > 0.05). Overall, a significant (*P* < 0.05) difference was observed among the five treatments, except for UT and W where the difference was not significant (*P* > 0.05).

To the best of our knowledge, no previous studies have been done on growth of *Listeria spp.* on crabsticks or any other surimi-based products. In our study, we showed that *Listeria* was able to grow albeit to a limited extent. However Rørvik et al. [23] studied the growth of *L. monocytogenes* in vacuum-packed, smoked salmon inoculated with *L. monocytogenes* at a high (2.7 log cfu/g) and low (0.8 log cfu/g) level and stored at 4 °C. After 4 weeks, the sensory attributes of the sample were still acceptable but this was not the case after 5 weeks. The main result was that *Listeria monocytogenes* multiplied to a high extent after 4 weeks, up to 3.7 log cfu/g in salmon inoculated with high level and 2.1 log cfu/g in the low level one. Also faster growth was observed in the salmon with lower inoculum. In the present study, slower growth was observed in the untreated sample with *L. innocua* increasing from 4.79 to 5.09 log cfu/g after 15 days.

Most other studies have tested the antimicrobial effect of essential oils against *Listeria spp.* in laboratory media. However, limited research has been conducted to validate the effectiveness of EO in a food system since the physical structure of food and the way in which it is stored can affect the effectiveness of essential oils. Campos et al. [24] indicated that the effective dose of essentials oils when applied in food should be higher than when tested in laboratory media. Hence, in the current study, we applied the highest concentration tested (0.5%) on crabsticks. It is worth noting that application of multiple hurdles such as antimicrobials; low temperature storage and vacuum-packaging are needed for optimal effectiveness of the treatments [15]. In the present study the use of lemon grass essential oil and extract were tested on crabsticks which were stored at a temperature of 4 °C to optimize the efficacy of the tested compounds. Low temperature storage alone will not appreciably inhibit the growth of Listeria spp since the latter is psychrotrophic with a minimum growth temperature of 1.7 °C [25]. However, application of essential oil together with low temperature storage can have a synergistic listeriostatic effect [24]. For example, clove oil was more inhibitory against *L. monocytogenes* in chicken frankfurters at 5 °C than at 15 °C [26].

Vacuum-packaging also extends the shelf-life of crabsticks by decreasing the level of oxygen present in the package and acting synergistically with refrigeration and antimicrobial treatment. Although in-package pasteurization of surimi-based products can eliminate all pathogens, contamination by *Listeria* is possible once the package is opened. Hence, a concerted action of pasteurization with vacuum-packaging and refrigeration are needed. It is also worth mentioning that since the pH of surimi ranged between 6.58 and 7, it can greatly favour the growth of *Listeria spp.*, which is known to grow over a wide pH range of 4.4–9.4.

To the best of our knowledge, there are no previous studies on the effect of LG on seafood products such as crabsticks. Cui et al. [19] tested the synergistic action of LG with cold nitrogen plasma against *Listeria* on pork loin. This synergy treatment enabled the use of a low concentration of EO (5 mg/ml for 30 min) thus not affecting the sensory attributes negatively. It also caused a 2.80 log cfu/g decrease in the *L. monocytogenes* count compared to the untreated sample. Unlike Cui et al. [19] who showed that lemon grass alone caused a decrease in the population of *Listeria* by 1.55 log cfu/g after 10 days at 4 °C; we recorded a population decrease of only 1.07 log cfu/g after 15 days using 0.5% lemon grass commercial essential oil.

Overall, we observed that 0.5% EO is a promising natural treatment to enhance the safety and quality of surimi. Other authors have tested different natural compounds on surimi including Chinese bayberry extract at 1 g/kg [27] as well as thyme EO at 0.8% and 1.2% [28] with varying degrees of efficacy.

### 3.3. Physicochemical analysis

#### 3.3.1. pH

The pH of surimi spanned the range 6.58–7.09 (Day 0) and 6.82–7.00 (Day 15) depending on the treatment (Table 2). The difference in pH observed over the storage period for each treatment was not statistically significant (*P* > 0.05). Hassan [29] also determined the pH of surimi made of different fish species and monitored the pH over an extended duration. Contrary to our finding, the author noted an increase in the pH from a minimum of 6.72 to a maximum of 6.93 after 15-day low temperature storage.

The mean pH of crabsticks for untreated, W- and ET-crabsticks had decreased after 15 days by 0.13, 0.09 and 0.12 pH units respectively whereas it had increased for EO and WBE by 0.2 and 0.24 respectively as shown in Table 2. Statistical analysis revealed that the treatment in question as well as the interaction between treatments and storage both affected pH of crabsticks significantly (*P* < 0.05).

**Table 2.** Mean pH of treatments on Day 0 and Day 15.

Days	UT	W	EO	WBE	ET
0	7.04 ± 0.11^abA^	7.09 ± 0.01^aA^	6.70 ± 0.06^bcA^	6.58 ± 0.04^cA^	6.97 ± 0.06^abA^
15	6.91 ± 0.05^abA^	7.00 ± 0.02^abA^	6.90 ± 0.05^abA^	6.82 ± 0.03^bA^	6.85 ± 0.04^bA^

Different lowercase superscript letters within each row indicate significantly different values (*P* < 0.05); Different uppercase superscript letters within each column indicate significantly different values (*P* < 0.05).

The slight decrease in pH of crabsticks treated with lemon grass compared to other treatments may be due to the acidic nature of the water-based extract and oil which have a pH of 3.50 and 5.84 respectively. Essential oils are more hydrophobic and thus more potent at acidic than at alkaline pH. In fact, the lower pH level is thought to enhance the dispersal of lipids of bacterial cell membranes; thus causing the cell wall to be less protected thereby enhancing the action of the essential oil [30]. On the other hand, samples left untreated or treated with water or ethanol underwent an increase in pH reaching 6.91 and 7.00 after 15 days although the difference was not significant (*P* > 0.05).

#### 3.3.2. Drip loss test

Water within meat products exists in bound, immobilized or free forms. Bound water molecules associate with electrically charged reactive groups of muscle proteins [31], while immobilized water molecules are attracted to the bound molecules in layers that become successively weaker as the distance from the reactive group on the protein becomes greater [32]. Free water refers to water molecules that are only held by weak forces [31]. Drip loss can be determined by quantifying the amount of free water lost in meat, chicken or seafood [33] and usually gives an indication of its juiciness [31].

Table 3 shows the decrease in weight of crabsticks subjected to different treatments over the 15-day storage period. The decrease in weight correlates with drip water loss occurring after 15 days. In this study, the extent of drip loss ranked in the order of UT (11.7%) > WBE (8.7%) ∼ W (8.6%) > ET (8.2%) > EO (4.5%) and could likely be due to osmosis or the decreasing water-holding capacity of the proteins during storage. Hassan [29] similarly noted that the moisture content of surimi made of different fish species underwent a significant decrease (*P* < 0.001) over a 15-day refrigerated storage.

**Table 3.** Average weight (g) of crabsticks from several treatments on Day 0 and Day 15.

Day	UT	W	EO	WBE	ET
0	15.21	15.31	15.28	15.37	15.24
15	13.43	13.99	14.59	14.03	13.99

#### 3.3.3. Colour measurement

The L* (lightness), a* (redness) and b* (yellowness) values of the five different treatments are shown in Table 4, 5 and 6 respectively. L* values of untreated crabsticks varied from 58.1–68.9. Spencer et al. (1992) also determined the L* values of surimi made of mackerel fish meat and reported L* values of 63.5–66.9. In descriptive terms, the author described the colour as dark and grayish. In our study, the treatments in question, the number of days of storage as well as the interaction between treatments and number of days of storage were found to have a statistically significant (*P* < 0.05) effect on the L* values of crabsticks. Lightness of crabsticks at Day 0 increased significantly (*P* < 0.05) with treatments W, WBE and EO relative to the control (UT) but decreased for ET-treated crabsticks. Moreover, L* values decreased over the 15-day storage for all treatments (UT, W, EO and WBE), but increased for ET-treated crabsticks. It is possible that ethanol resulted in the denaturation of proteins conferring a whiter colour to the crabsticks. The decrease in L* values over storage could have been due to the occurrence of browning reactions during storage. For crabsticks treated with EO or WBE, the darker colour could have been due to the inherent colour of the essential oil or extract of lemongrass.

**Table 4.** Effect of treatments on the L* values of white side of crabsticks at Day 0 and Day 15.

Time (days)	Treatments				
	UT	W	EO	WBE	ET
0	68.9 ± 0.17^dA^	74.4 ± 0.22^cA^	79.1 ± 0.30^aA^	75.5 ± 0.50^bA^	64.9 ± 0.21^eA^
15	58.1 ± 0.18^cB^	73.4 ± 0.13^bB^	75.3 ± 0.24^aB^	73.8 ± 0.17^bB^	75.4 ± 0.17^aB^

Different lowercase superscript letters indicate values within the same row that were significantly different; Different uppercase superscript letters indicate values within the same column that were significantly different.

Surface colour a* and b* values of treated crabsticks were also found to be higher compared to their untreated counterparts. This could be due to acquisition of a more pronounced reddish or yellowish colour. However, both a* and b* values decreased significantly over the 15-day storage possibly due to fading.

**Table 5.** Effect of treatments on the a* values of red side of crabsticks at Day 0 and Day 15.

Time (days)	Treatments				
	UT	W	EO	WBE	ET
0	26.7 ± 0.52^cA^	29.0 ± 0.15^bA^	30.6 ± 0.26^aA^	28.3 ± 0.24^bA^	31.1 ± 0.27^aA^
15	26.7 ± 0.07^cA^	27.2 ± 0.24^bB^	29.4 ± 0.74^aB^	25.6 ± 0.15^dB^	29.0 ± 0.15^aB^

Different lowercase superscript letters indicate values within the same row that were significantly different; Different uppercase superscript letters indicate values within the same column that were significantly different.

Many plant extracts have a dark colour and they can impart a negative colour change in food systems depending on their concentration and nature of the food matrix [27]. Thus a colour measurement apparatus must be used to evaluate the effect.

Surimi is normally white in colour since it is washed thoroughly to remove muscular pigments, meat lipids and blood that lead to darkening [34]. Natural dyes such as E120 (carmine) and E160C (paprika) are then added to one surface to provide colour to the crabstick causing the product to resemble the cooked natural crab [34]. Carmine normally gives a red hue, while paprika provides an orange hue in general. Carmine contains the coloring agent carminic acid molecule (about 50%), which is extremely soluble in water. The decreasing a* value during storage could be attributed to the high moisture content in surimi causing the red colour to wash off and fade [35]. Paprika oleoresin is not water-soluble; however it is quite unstable against oxidation, light, heat and storage compared to carmine colorant. In fact, even storage of paprika oleoresin-colored food products in dark and cold conditions can cause fading [35]. These observations may be the reason accounting for the slight decrease in a* and b* values, that is redness and yellowness of all the treatments. Moreover, the paprika colour is known to change from pale yellow to reddish orange with changes in pH [35]. The L* value decreased slightly in almost all the treatments but no traces of carmine bleeding was identified.

**Table 6.** Effect of treatments on the b* values of red and white sides of crabsticks at Day 0 and Day 15.

Time (days)	Treatments									
	UT		W		EO		WBE		ET	
	Red side	White side	Red side	White side	Red side	White side	Red side	White side	Red side	White side
0	33.1±0.20^eA^	14.3±0.34^hA^	36.1±0.28^bcA^	15.4±0.15^gA^	36.7±0.19^abA^	19.0±0.22^fA^	35.4±0.22^cdA^	14.5±0.24^hA^	36.9±0.21^aA^	15.7±0.18^gA^
15	33.1±0.14^cB^	13.8±0.14^fA^	33.8±0.18^bB^	14.5±0.22^eB^	35.0±0.22^aB^	13.8±0.20^fB^	30.2±0.23^dB^	13.3±0.21^fB^	34.5±0.18^aB^	14.9±0.15^eB^

### 3.4. Sensory evaluation

**Figure 2.**
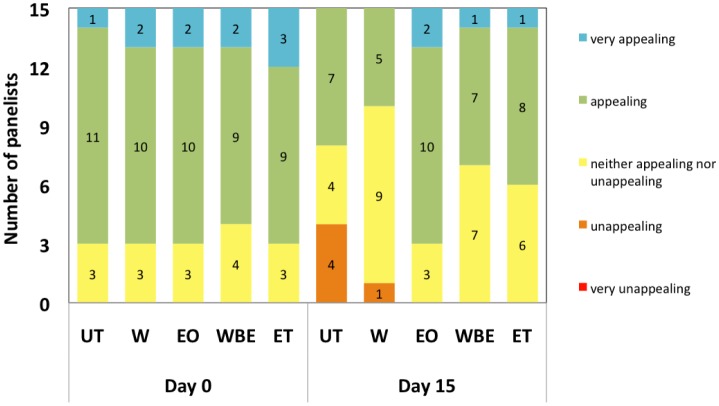
General appearance of samples at day 0 and day 15.

The general appearance or appeal of crabsticks did not vary significantly (*P* > 0.05) with treatments although storage duration had a significant (*P* < 0.05) effect on the appearance of treated and untreated surimi. 70–80% of the panelists found the EO- or WBE- treated and untreated crabsticks appealing or very appealing on Day 0 (Figure 2). However, after 15 days, only 47% and 53% of panelists found the untreated and WBE-treated crabsticks appealing to very appealing (Figure 2). However, EO-treated crabsticks were still well received, with 80% of panelists finding the sample appealing or very appealing. This could be because of the colour conferred by the essential oil. The finding for untreated crabsticks is in agreement with observations made by Hassan [29] who also observed that the appearance scores of surimi made of tilapia, shark and nemipterus fish meat decreased after 15-day storage.

**Figure 3.**
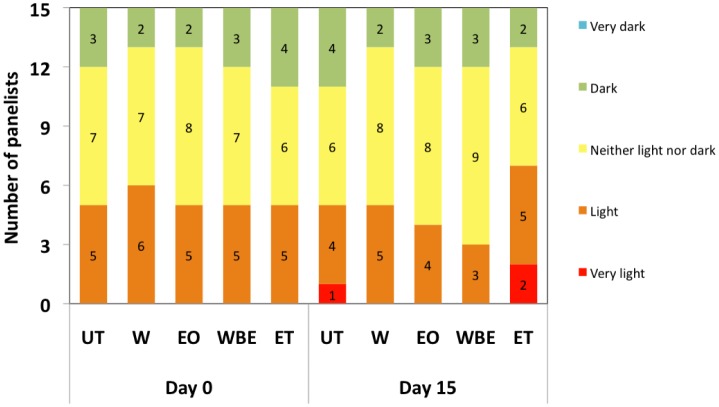
Colour density of samples at day 0 and day 15.

According to Park et al. [36], gel colour is an important characteristic of surimi. In this study the colour density of crabsticks was not significantly (*P* > 0.05) affected by treatment, storage duration, or the interaction of treatment and storage duration. However, 7 out of 15 panelists (47%) reported that the colour of the samples treated with ET became lighter compared to the other treatments while 12 out of 15 (80%) reported an increase in colour density for WBE-treated crabsticks (Figure 3). People have a certain expectation concerning the colour of food products that they are consuming and if the food's colour is unattractive, they may find the products unappealing overall [37]. The consumer may not want to taste the food product thus colour is an important determinant since it is the first element noticed in the appearance of a product [37]. Although the EO and extracts have a straw-like to brown colour, their application did not adversely affect the overall appearance of the treated crabsticks after 15 days.

**Figure 4.**
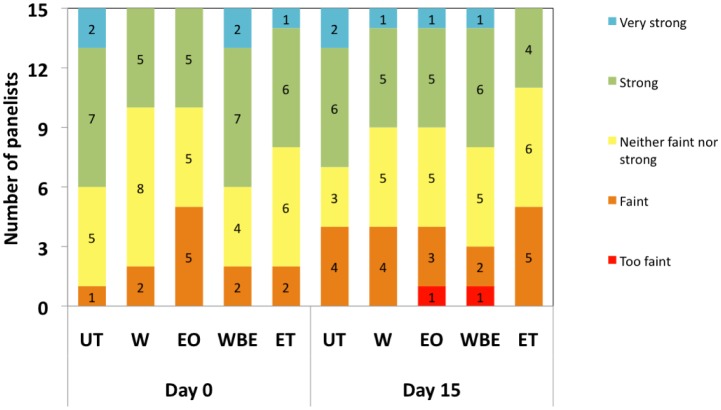
Aroma of samples at day 0 and day 15.

The different treatments, duration of storage as well as the interaction between treatments and day of storage did not significantly (*P* > 0.05) affect the aroma of crabsticks. On the contrary, Hassan [29] noted that the odour intensity of surimi decreased significantly (*P* < 0.001) with time. In the current study, 60–67% of panelists thought that untreated crabsticks had a strong to very strong unpleasant odour at Day 0 and Day 15, and described the smell as “fishy” (Figure 4). This is contrary to our expectations, as surimi is a bland material devoid of any fishy odour. Since surimi is made after repeated washing during manufacturing, most of the odour-imparting compounds, pigments, water soluble proteins and other undesirable materials are removed [38]. It is possible that during storage some biochemical reactions occur in the muscle, thereby contributing some undesirable off-odour to the meat Hassan [29]. For crabsticks treated with EO, 33–40% of panelists perceived a strong to very strong pleasant odour, which they described as “lemongrass-like” (Figure 4). Similarly for crabsticks treated with WBE, 60% and 47% of panelists identified a strong to very strong “lemongrass-like” aroma at Day 0 and Day 15 respectively. It is likely that the intensity of the aroma of the WBE extracts decreased during extended storage.

**Figure 5.**
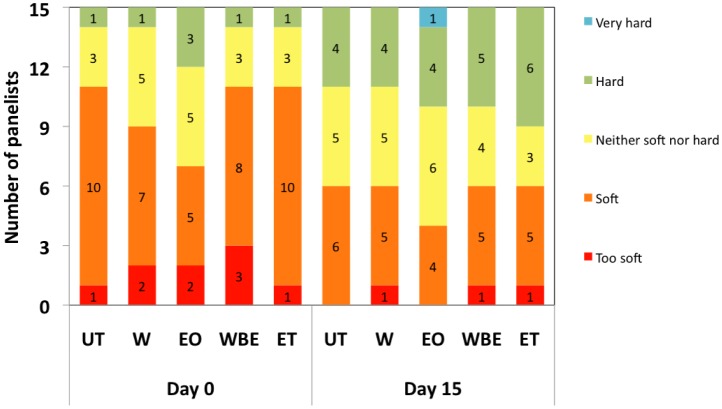
Texture of samples at day 0 and day 15.

Texture is another important attribute contributing to the acceptability of the consumer. The texture scores of crabsticks were significantly (*P* < 0.05) affected by storage although treatment and the interaction of treatment and storage did not have any significant (*P* > 0.05) effect. A general decrease in softness of all samples was observed by most of the panelists (Figure 5). For instance, 67% of panelists perceived ET-treated crabsticks as “soft” at Day 0 compared with only 33% at Day 15 (Figure 5). Our findings are congruent with those of Hassan [29] and Lee and Toledo [39] who attributed the increased toughness (or hardness) of surimi during storage to decreased moisture content leading to lower gel resilience and cohesiveness. The preference for texture varies among people; a softer texture may be preferred by some and a firmer or harder texture by others. Texture of surimi-based products such as crabsticks has a tendency to be softer than meat as less connective tissue is present with weaker cross-links between collagen molecules [38].

Moreover, since crabsticks are made of several sheets of surimi, it is even softer. Texture may be better assessed by mouth-feel when the food is chewed but in this study it was evaluated with the fingers by applying pressure on the crabstick.

**Figure 6.**
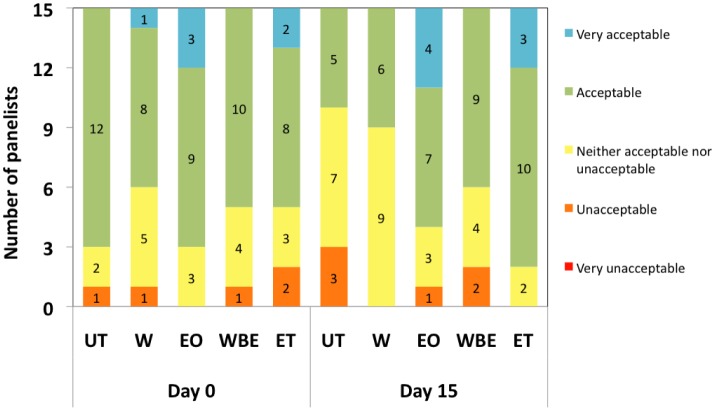
Overall acceptability of samples at day 0 and day 15.

With regard to overall acceptability, we observed an inverse relationship between the acceptability scores of untreated crabsticks and days of storage (Figure 6). This observation is congruent with findings of Hassan [29] who also noted that the overall acceptability scores of surimi meat made of common carp, tilapia, shark and nemipterus decreased during storage. Except for untreated crabsticks, all other samples were found to be still acceptable even after the 15-days storage. 60%, 73% and 86% of panelists found WBE, EO and ET-treated crabsticks to be acceptable to very acceptable. Panelists found ET to be slightly more acceptable than their treated counterparts probably due to its more appealing colour and aroma.

To the best of our knowledge, no previous studies have been carried out on the sensory attributes of lemon grass on crabsticks or other surimi-based products. However, quite a few studies have tested the effect of essential oils on the sensory attributes of seafood. For instance, Kerekes et al. [40] showed that essential oils (0.05% Oregano oil) applied on cod fish fillets gave a different but pleasant flavor. Also, 0.4% Oregano oil applied on rainbow trout fillets did not significantly affected the sensory attributes (odour, taste) of the products when kept at a refrigerated temperature of 4 °C [41]. Another study showed that rainbow trout's treated with 1%, 1.5% and 2% cinnamon oil was well received by panelists [42]. In yet another study, thyme essential oil was added to vacuum packed trout fillets and no unpleasant effect was observed concerning the taste, odour, texture and appearance when stored at a cold temperature [41]. Thyme oil also gave a pleasant odour to sea bass fillets and extended its shelf-life [41]. Findings of these studies are in agreement with our observation that EO-treated surimi were found to be acceptable or highly acceptable by the majority of panelists.

## 4. Conclusion

The demand for food without any synthetic preservatives is continuously increasing. Current study showed that lemon grass treatments could effectively reduce the counts of *Listeria monocytogenes* on crabsticks without causing any negative effect on its sensory attributes. Lemon grass essential oil demonstrated a more effective treatment compared to its water-based extract, thus lemon grass essential oil may be applied to food systems in order to increase their shelf-life. Further experiments of LG essential oil can be extended to other seafood products such as fish fillets. The effect of the oil on the taste of the product must also be tested and thus find a minimum concentration that will prevent microbial growth and also satisfy consumer preferences. Using natural products as antimicrobials can not only reduce the harmful impact of synthetic preservatives on human health but also be more cost-effective. The organoleptic impact of plant essential oils in food products currently restricts their usage but several approaches like edible coating, nano-encapsulation, or combination of EOs with other preservatives can represent a solution to this problem.
